# Consume, Modify, Share (CMS): The Interplay between Individual Decisions and Structural Network Properties in the Diffusion of Information

**DOI:** 10.1371/journal.pone.0164651

**Published:** 2016-10-31

**Authors:** Hila Koren, Ido Kaminer, Daphne Ruth Raban

**Affiliations:** 1 Department of Information and Knowledge Management, University of Haifa, Haifa, Israel; 2 Department of Physics, Massachusetts Institute of Technology, Cambridge, MA, United States of America; Nankai University, CHINA

## Abstract

Widely used information diffusion models such as Independent Cascade Model, Susceptible Infected Recovered (SIR) and others fail to acknowledge that information is constantly subject to modification. Some aspects of information diffusion are best explained by network structural characteristics while in some cases strong influence comes from individual decisions. We introduce reinvention, the ability to modify information, as an individual level decision that affects the diffusion process as a whole. Based on a combination of constructs from the Diffusion of Innovations and the Critical Mass Theories, the present study advances the CMS (consume, modify, share) model which accounts for the interplay between network structure and human behavior and interactions. The model's building blocks include processes leading up to and following the formation of a critical mass of information adopters and disseminators. We examine the formation of an inflection point, information reach, sustainability of the diffusion process and collective value creation. The CMS model is tested on two directed networks and one undirected network, assuming weak or strong ties and applying constant and relative modification schemes. While all three networks are designed for disseminating new knowledge they differ in structural properties. Our findings suggest that modification enhances the diffusion of information in networks that support undirected connections and carries the biggest effect when information is shared via weak ties. Rogers' diffusion model and traditional information contagion models are fine tuned. Our results show that modifications not only contribute to a sustainable diffusion process, but also aid information in reaching remote areas of the network. The results point to the importance of cultivating weak ties, allowing reciprocal interaction among nodes and supporting the modification of information in promoting diffusion processes. These results have theoretical and practical implications for designing networks aimed at accelerating the creation and diffusion of information.

## Introduction

Complex networks are characterized by emergence and self-organization dynamics, in which local decisions and interactions accumulate into system-level phenomena. The diffusion of information in complex networks is propelled by decisions made at the local level of individual nodes to consume and share the information, but, as identified by Christakis and Fowler [1, 2] the overall outcome depends on structural properties. This work attempts to study the interplay between the decisions taken on the node basis and structural properties such as directionality and tie strength.

Widely used Information diffusion models often use simplifying assumptions in order to describe complex phenomena. For example, models such as Susceptible-Infected-Recovered (SIR) [3] or Susceptible-Infected-Susceptible (SIS) [4] assume that information diffuses similarly to virus contagion dynamics and that mere exposure suffices for information transfer and adoption. Recent research with SIR and SIS models in the contexts of multi-layered networks [5] suggests that contagion corresponds to a two layer network: one layer where awareness dynamics evolve (online social contacts) and another where the epidemic process spreads (physical contacts). The importance of studying the spread of information in parallel to the spread of disease comes from the interest in using information to delay epidemic thresholds. In this context it is vital to explore the unique threshold in information diffusion processes.

We suggest that information diffusion involves a process that is affected by a combination of three factors: (1) the *actors* comprising the network; (2) *content*, or properties of the information itself; and (3) the underlying *network structure*. By combining all three, the present work offers comprehensive understanding of the dynamics of information diffusion.

Regarding the actors, we present a two-stage decision making process that involves volition: the decision to consume information, and upon consumption, whether to pass it on. Regarding content, actors have the option to transmit the information "as is" or, to modify it *before* passing it on. In this context, we present the concept of *reinvention* adapted from the Diffusion of Innovations (DOI) Theory. Reinvention, i.e. modification of information, is considered on two levels: as an attribute of the information itself, allowing an investigation of whether it enhances or diminishes the value of information as it spreads in the network; and as a behavior performed by nodes in the network. The dynamics involved in modification activity shed light on whether the act of modifying information has an accelerating or decelerating effect on diffusion. The third factor of network structure relates to whether the network is directed (supports asymmetrical communication patterns) or undirected (supports symmetrical communication patterns), as well as to the tie strength between nodes.

The present work offers an agent based model that accounts for volition and possible changes information undergoes throughout the diffusion process. The model combines theoretical constructs from the Diffusion of Innovations Theory [6] and Critical Mass Theory [7] that address the conditions necessary for activities to start and become self-sustained. From the Diffusion of Innovations we adopt the concepts communication channels (directionality), communication proximity (tie strength), individual behavior (modification of information) and the inflection point. From Critical Mass Theory we build upon the concept of production functions manifested in group processes. The model reflects volition by considering individual decisions to consume, modify, and share information and seeks to find the emergent effect of these local preferences with special attention to the tie strength between nodes.

The model is applied on the social graph of three distinct networks: DBLP network of research scientists, US Patent citation network and Wiki Talk network of conversation. These networks share the purpose of information dissemination, but, they differ in the communication patterns they support: while the DBLP is an undirected network that allows reciprocal communication, US Patent and WikiTalk are directed networks supporting one-way communication. The question motivating this research is: How does the interplay between individual decisions and structural network properties such as direction and tie strength affect the diffusion of information in networks?

Our findings imply that the modification of information has a varying effect throughout the diffusion process. While having a negative effect on attaining the inflection point, it has a positive effect on overall diffusion, as modified information propagated in the network longer, reaching a higher percentage of nodes positioned further in the network than information that is shared "as is". Furthermore, our findings indicate that the greatest effect of modifications on diffusion is found when communication is reciprocal (undirected) and when information is shared via weak ties. These findings question and fine-tune Rogers' diffusion model and traditional information contagion models.

The rest of the paper is structured as follows: we begin theory development by presenting known models of information diffusion and explain the need for accommodating the human aspects of volition and modification. We then describe the main constructs of the Diffusion of Innovations Theory and the Critical Mass Theory and introduce the manner by which tying those two theories together leads to the consume-modify-share (CMS) model for information diffusion. Next, we describe the research questions, data used, method and the results obtained. The paper continues with a discussion that outlines the research contribution to theory and concludes with addressing limitations and identifying areas for future research.

## Theory Development

### The Need for a Contemporary Model for Information Diffusion

Widely used information diffusion models such as Independent Cascade Model [8, 9]; Continuous Time Independent Cascade Model [10]; Susceptible Infected Recovered (SIR) Model [3]; Susceptible Infected Susceptible Model (SIS) [4] and Rumor Spreading Model [11] share the following attributes: (a) information is synonymous to disease, (b) mere exposure to information, like exposure to a virus, is enough for "contagion", i.e. information diffusion, (c) the population is uniformly mixed where all members are equally susceptible to the "disease". These models are "sender centered" where each sender independently influences its neighbors with some probability.

Other models, such as the Threshold model [12] assume that members of the population are not uniformly affected–the decision to adopt depends on whether the number of a member's adopting neighbors exceeds some personal threshold. This approach turned attention from the sender to the receiver of information, and from global ratios to combining global and local structural properties such as influencers (hubs) and their effect on the size and likelihood of a cascade to occur.

Threshold models gave way to a line of research dealing with maximization calculations and network influence models focusing on how to effectively trade-off the cost of seeding (i.e., identifying and targeting the most influential members of the population) and the size of the cascade generated. Contagion and threshold models assume information diffuses from its originator in a multi-step process that gradually reaches a large number of individuals.

Recently, the study of complex networks has distinguished between interacting network layers, giving rise to research on multi-layered networks [5, 13, 14, 15]. Multi-layered networks describe social interactions that occur at different contexts or categories, each forming a layer that supports different dynamic processes that interact. For example, disease contagion corresponds to a two layer network, a layer of online social contacts transmitting information and generating disease awareness, and a layer of physical encounters where the epidemic process spreads. Analyzing these layers allows exploring the interplay between spreading of the awareness and the epidemic infection itself, and their interaction with mass media.

Interesting research on SIR and SIS modeling of epidemics in complex networks indicates that the existence of a critical threshold is correlated with the topology of the network [16, 17]. While the research on epidemiology focuses on finding inhibiting mechanisms (high threshold) for the spread of contagion, the study of information transfer focuses on mechanisms that promote its diffusion (low threshold).

Nevertheless, epidemiological and threshold models, when adapted to model the diffusion of information, do not capture the unique properties of information such as: (1) information is not synonymous with disease and therefore exposure to it is not enough for it to spread. Although senders of information can send information indiscriminately (such as junk mail), receivers of new information are faced with two decisions that existing models fail to address: the decision whether to consume the information, and upon consumption, the decision whether to pass it on to others. Explicit retransmissions are vital for information to spread and reach exposure to a critical mass of people, (2) in most models, the entity being spread (information, virus) is assumed to be invariant during the diffusion process. Yet in realistic situations, information is prone to modifications as it diffuses. Modifications can be enhancements or deletions, intentional or erroneous. They may add or diminish value from information [18, 19]. (3) Existing models are either sender or receiver centered. Our model accounts for the relation between senders and receivers by combining tie strength.

#### What effects the individual decision to consume and share information?

Information diffuses by explicit retransmissions. These retransmissions are affected by the decision to consume (read/ view) the information, and the decision whether to share it with others. Literature addresses these decision phases by looking at properties of the *content*, by *sender/receiver motivations* and by *network structure*. From the *content* perspective, researchers have identified the following influences on the decision to consume and share information: emotional appeal [20, 21], resonance [22], humor [23, 24], salience [25, 26] and usability [27].

Information sharing is a complex, multidimensional phenomenon affected by behavioral, social and technological factors [28]. Regarding *motivation*, sharing information is found to be affected by altruistic or self- enhancement motives–to appear knowledgeable [27] and increase status [10]. Fehr, Korchsteiger & Riedle [29] found that people share information to generate reciprocity. Finally, according to Berger and Milkman [20], sharing information serves as means to communicate identity.

*Network structure* impacts the spread of information within it [1, 2, 7]. In this work, we relate to two structural properties: direction of the network (a global property) and tie strength (a local property). Regarding directionality: A *directed* network is a set of nodes connected by edges, in which the edges are directional, i.e., information flow is asymmetric. For example, in Twitter "following" others does not imply that the connection is reciprocal. According to Barabasi [30] directed communication limits the navigability of information in the network, and the extent of its reach. An *undirected* network is a set of interconnected nodes, where all edges are bidirectional: when a link is formed in an undirected network it facilitates bidirectional interaction between any two nodes, as in Facebook. This symmetry doubles the opportunities for sharing information as compared to directed networks where interaction is unidirectional. In this work we analyze two directed networks (Wiki Talk and US Patent) and one undirected network (DBLP).

Ties between nodes in a network vary in their intensity and intimacy, which is described by tie strength. Tie strength can be measured by communication reciprocity [31], recency of communication [32] and interaction frequency [33, 34]. Tie strength is associated with *content*, the *speed* at which information diffuses in the network, and its overall *reach* as explained below. Weak ties, more than strong ones, tend to play the role of transmitting unique, novel and non-redundant information across otherwise largely disconnected segments of complex networks, thereby increasing the diversity of information propagated in the network [34, 35]. Relating to information reach and speed, Nahon and Hemsley [36] found that while strong ties help distribute information faster; weak ties are better at spreading it further. Zhao, Wu and Xu [37] found that weak ties help overcome the trapping of information in local clusters.

The next section explains the relevance of modification to the diffusion process through the lens of the Diffusion of Innovations Theory. The addition of elements from the Critical Mass Theory aids in building the CMS model, which accounts for content as well as for the sender/receiver and structural aspects.

#### The relevance of modifications to diffusion

Information is malleable, as can be seen by memes, mashups, and progress of scientific research, versions of news items and other examples. Modification is often intentional, as part of a creative and personalized process. The Diffusion of Innovations Theory refers to such changes as "reinvention" which is defined as modifying an innovation by a user in the process of adoption and implementation [38]. Modifications are measured by the degree to which elements of the innovation are similar to, or different from the core elements of the innovation [39]. The concept of reinvention addresses two notions we believe to be important for understanding diffusion of information: (a) information is prone to modifications, as it diffuses (b) sharing information serves self-presentation purposes by gaining status and recognition [40] and as a form for expressing creativity, crafting self-identity and building social relationships through creative processes [37].

Nowadays, when information consumers are able not only to forward information but also to annotate, appropriate, and recirculate content with relative ease [41], we propose the modification of information, as an unexplored, yet relevant variable to diffusion studies. Modifying information relates to motivational and content attributes involved in diffusion. Regarding the first, modifications serve as means to display creativity, to enhance of one’s image and build social ties. In terms of content, the opportunity to modify content draws engagement and carries the potential of spreading valuable information throughout the network as modifications imply that information undergoes an evolutionary process.

Modifications are thus considered on two levels: as an attribute of the information itself, allowing an investigation of whether modifications enhance or diminish the value of information as it spreads in the network; and as a behavior performed by nodes in the network. In this manner, the dynamics involved in "modification activity" shed light on whether the act of modification has an accelerating or decelerating effect on diffusion.

### Diffusion of Innovations Theory & Critical Mass Theory

The Diffusion of Innovations theory (DOI) explains how innovations, ideas and technologies, are taken up in a population. Critical Mass Theory (CMT) predicts the probability, extent and effectiveness of group actions in pursuit of a collective good [7]. Both theories aim to provide an explanation regarding the conditions when activity starts and becomes self-sustained. Both theories offer a comprehensive description that combines individual and group characteristics, the diffusing good's attributes, and the supposition of sequential interdependence in which individuals make independent decisions, but past decisions made by others are known to them and influence them. Both theories state that heterogeneity of members in the social system in terms of resources and interest levels (CMT) and innovativeness levels (DOI) is necessary for the emergence of the initial participants starting the diffusion process. We suggest that when information is the innovation that diffuses in a network, combining constructs from both theories allows a deeper understanding regarding the dynamics associated with critical mass formation and overall diffusion as outlined next.

DOI theory defines diffusion as the process by which an innovation is communicated through certain channels over time among members of a social system. Four key elements are identifiable in every diffusion research study: the social system in which the innovation takes place, the communication channels of that social system, time, and the innovation's attributes [42]. Each element is described with adaptations from CMT.

The *social system* in which innovation takes place is defined as a set of interrelated units that are engaged in accomplishing a common goal. This sharing of a common objective defines the boundary within which an innovation diffuses. In this work, we study three social systems–three distinct networks dedicated to the objective of disseminating innovative information.

The *channels of communication* among members of a social system determine the nature of information exchange and the effect of its transfer. In this research the channels of communication are determined by the directionality of the links and communication proximity, a term synonymous with Granovetter's [34] distinction between weak and strong ties. The model we introduce incorporates weak and strong ties allowing us to further determine their effect on the diffusion process in the three networks analyzed.

According to DOI, the *time* element is associated with two dimensions: innovativeness (individual level) and the rate of adoption (system level). Rogers classifies individuals in a social system into five adopter categories according to their innovativeness level, i.e., the degree to which they are relatively earlier in adopting new ideas than other members. Each adopter category is associated with personality traits, communication behavior and socioeconomic status. The five categories are: Innovators, which comprise 2.5% of the social system, Early Adopters (13.5%), Early Majority (34%), Late Majority (34%) and Laggards comprising 16% [38]. The measure of innovativeness is based upon the relative time at which an innovation is adopted.

The rate of adoption is inferred by plotting the cumulative number of individuals adopting a new idea over time. The resulting distribution is an S-shaped curve indicating progressive diffusion through the five categories. Critical mass occurs at a point in time in which the rate of the adoption is fastest, i.e. the number of new adopters is increasing most rapidly. This point, referred to as the *inflection point*, occurs when about 16% of the individuals have adopted an innovation. According to the DOI, once the adoption of an innovation reaches an inflection point, further diffusion becomes self-sustained [38].

Rogers equated critical mass with the occurrence of an inflection point; however, we find the definition of the critical mass as described in CMT and its description of production functions, as a substitute for the S-shape distribution, to be of relevance to the diffusion of information. CMT does not define the "critical mass" as an absolute percentage. Rather, it relates to a "small segment of the population that chooses to contribute to the collective action thus creating conditions for the majority to join leading to the achievement of the collective good". In social settings, the critical mass, as described in CMT, comprises the initial seed nodes that start the diffusion process.

**Fig 1:**
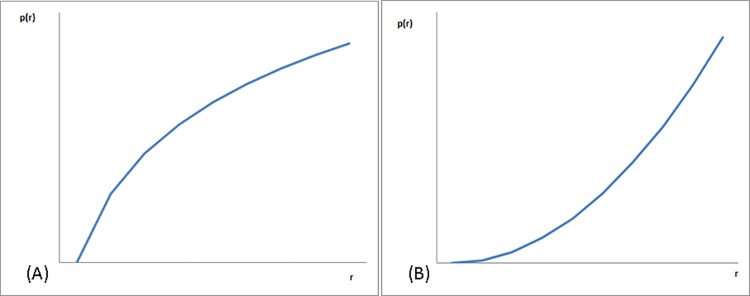
Production functions in critical mass formation. (A) Decelerating production function. (B) Accelerating production function.

In this research, the success of the initial set of nodes to influence diffusion is determined by their ability to draw repeated participation of subsequent nodes in the act of sharing information. This ability, according to CMT, is a consequence of two distinct social dynamics described as "production functions": the first describes situations in which the earliest contributors have the greatest effect on achieving the public good, and subsequent contributors have progressively less effect. This situation is referred to as a *decelerating production function* (PF) (Fig 1A). In the accelerating situation, initial contributors have negligible effects on achieving the collective good, and subsequent contributors yield an increasing effect (Fig 1B). In a decelerating production function, interdependence between nodes is negative: each contribution makes others' subsequent contributions less valuable, and thus less likely. For example, adding a book review to an existing review repository is expected to follow a decelerating function. In an accelerating production function, interdependence between nodes is positive: an early contribution makes subsequent ones more worthwhile and thus, more likely. For example, in application stores, early raters are important for starting the process, and later raters add more value as the rating becomes more significant when it is based on a large number of raters. The decelerating function is self-limiting. The accelerating production function is self-reinforcing resulting in considerable diffusion.

In the study of information modification and its effect on diffusion, the concept of production functions allows a deeper inquiry into the dynamics associated with attaining an inflection point and the effect of modifications on overall diffusion. In order to simulate decelerating and accelerating production functions we operationalized modification as a constant or relative change in the value of information, respectively. These value change schemes are elaborated in the Model Description section below. This differentiation allows us to better examine the dynamics associated with reaching the inflection point and the interplay between structural network properties such as directionality and tie strength and individual behavior of performing modifications involved in the process.

### Research Proposition Development

Combining constructs from the DOI: communication channels (directionality), communication proximity (tie strength) and individual behavior (modification) with group processes manifested by the production functions from the CMT leads to our research question:

*How does the interplay between individual behavior and structural network properties affect the diffusion of information in networks?* The question is addressed in three stages: up to and following the inflection point and with regard to the cumulative value of information that diffuses throughout the networks.

When a link forms in an undirected network it facilitates bi-directional interaction between any two nodes. This doubles the opportunities for sharing information as compared to directed networks were interaction is uni-directional, therefore, we explore the following propositions:

**P1 (a)** Individual behavior (modification) affects the realization of the inflection point.
**(b)** The effect of individual behavior on reaching the inflection varies with network directionality.
  Messages spread faster in strong tie clusters, but need weak links to span network holes and reach farther into a wider network [30, 38, 43, 44]. Thus we propose:
**(c)** The effect of individual behavior (modification) on reaching the inflection point differs with tie strength

As previously mentioned, DOI theory states that once the adoption of an innovation reaches an inflection point, further diffusion becomes self-sustained [38]. We observed that self-sustainability of the diffusion process is determined earlier than the appearance of the inflection point, as will be shown in the Results section. This finding led us to a deeper inquiry into the concept of sustainability.

Sustainability is the degree to which an innovation continues to be used over time [38: p183]. Furthermore, Rogers points out that "unless the client feels so involved with the innovation that they regard it as theirs, it will not be continued over the long term". Modification, in this context, manifests a level of involvement. CMT's production functions offer a rich explanation regarding the dynamics involved. According to CMT, accelerating and decelerating social dynamics determine the extent and sustainability of the diffusion process. In this light, we turn attention to the sustainability of the process beyond the inflection point, and ask: *How does the interplay between individual behavior (modification activity) and structural network properties affect the information diffusion process in terms of reach and sustainability?* We propose:

**P2:** The influence of individual behavior on the reach of information is affected by (a) network directionality and (b) tie strength.
**P3:** The influence of individual behavior on sustainability of the diffusion process is affected by (a) directionality of the networks the (b) tie strength between nodes sharing information.

Lastly, Rogers describes the *innovation's attribute* as the fourth element involved in diffusion research. When information diffuses it is susceptible to change which affects its perceived value. The analysis of production functions allows determining whether modifications enhance the value of information that spreads in the network and if so, which modification type contributes to the process. This leads to an additional fine tuning of the research question: *How does the interplay between individual behavior (modification) and structural properties affect the overall value of information that spreads in the network?* We propose:

**P4:** The contribution of modification to the value of information that diffuses in the network is higher in undirected networks than in directed ones.
**P5:** The contribution of modification to the value of information that diffuses in the network is higher when shared via weak ties than when shared via strong ties.

## Data

In order to test information flows in a realistic topology, data is comprised of three actual networks harvested from the web: DBLP network of research scientists, Wiki Talk network, and the US Patent citation network. These networks provide a diverse source of data as they vary in properties such as size, mean degree and direction.

The **DBLP** computer science bibliography, retrieved from the SNAP website [45], is a co-authorship network where two authors are connected if they published at least one paper together; therefore, it is an *undirected* network.

**Wiki Talk** (Wiki): retrieved from SNAP [45], is a network containing all the users and discussions from the inception of Wikipedia till January 2008. Nodes in the network represent Wikipedia editors and a *directed* edge from node *i* to node *j* represents that user *i* edited a talk page of user *j* at least once.

**US Patent citation network** retrieved from SNAP [45] includes all citations made by U.S patents granted during 1975–1999, totaling 16,518,948 citations. As the Patent network is highly segmented, we included in our data the largest portion of the network that is connected, which comprises of 26,925 patents. Based on the nature of citing, this network is *directed* both in the direction of the flow of information and temporally.

As seen in Table 1, the size of the networks ranges between tens of thousands to millions of nodes, with varying nodes-to-links ratios.

**Table 1:** Data Description.

Network	# Nodes	# Links	Mean degree	Direction	Link formed	Tie strength
DBLP	317,080	1,049,866	6.6221	Undirected	Co-authoring	# of co-authors
WikiTalk	2,394,385	5,021,410	2.0972	Directed	Editing	# of editions
US Patent	26,925	51,694	1.9199	Directed	Citation	# of citations

## Method

Agent-based modeling (ABM) is a computation analysis tool used for simulating system dynamics when the system consists of multiple autonomous and interacting components, or "agents". Each agent follows a set of rules that can be either identical for all agents (homogenous) or different from agent to agent (heterogeneous). ABMs are often applied to validate individual-level configurations by reproducing patterns that result in the system level.

In the social sciences, ABM has become increasingly popular as it enables to build models representing individual entities and their interactions. By offering the possibility of modeling individual heterogeneity and an explicit representation of agents' decision rules, these models represent multiple scales of analysis and the emergence of macro structures from individual action [33]. ABM's are employed in fields such as social sciences [32], information studies [46], environmental research [47] and marketing [8].

The present model incorporates the decisions to Consume, Modify and Share information; therefore, we name it the CMS Model. CMS is an agent-based model constructed using MATLAB (version 7.12). The model is based on discrete step iterations to demonstrate the influence of modifications on the process of critical mass formation and the overall diffusion of information. The CMS model incorporates theoretical constructs based on prior literature [6, 7, 48], where variables in the model are given a certain value or probability.

### Model Description

Nodes in the network were assigned various probabilities for each of the following states:

1. before receiving information
2. received information but did not consume
3. consumed without modifying, then did not share
4. consumed and shared without modification
5. consumed and modified but did not share
6. consumed, modified, and shared

**Fig 2:**
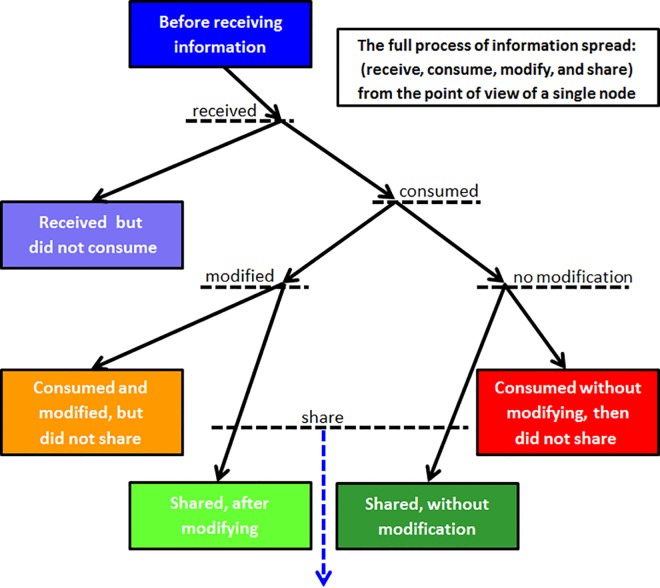
CMS Model of information flow with assigned probabilities

Fig 2 depicts the application of the formulas to the various states of the nodes including some of the variables and probabilities explained below.

#### Simulation procedure

The model was run on the flow of information between nodes without performing modification and then again with modification, in order to isolate the unique effect of information modification. After simulating different starting points, peripheral and central nodes, we chose the option that showed the greatest variance in results: the weakest neighbor of the most connected hub.

Each starting node received a standard assignment of value of information set to V = 1 at the beginning of the experiment. Modification was either constant (AM) or relative (RM), corresponding, respectively, to decelerating or accelerating production functions. Each node modified the information independently, yet the simulation allowed the study of the collective outcome.

The dynamics start to evolve by the starting node’s neighbors who are the new receivers of information. Information arrives with a certain value assignment according to the node state detailed above. Each new receiver node "decides" what to do with incoming information based on the probabilities assigned to various possible actions. In particular, each node decides whether to consume, modify, and then whether to share the information with its neighbors (Fig 2).

Each decision phase constitutes one experiment iteration. The new state obtained leads to continuing the sharing of information according to the model rules. Modifications occur upon consumption of information before the decision to share. The process is repeated by finding the new receivers. Iterations repeat until final resolution when information cannot continue to flow between nodes, and the network reaches its final state. At this point, we assess whether critical mass was reached, the magnitude of collective outcome, and the degree of spread.

The number of iterations needed before convergence depends on the network diameter and density. In order to get representative results and verify sufficient simulation results to extract average measures, we simulated the full process of information flow and repeated the full experiment thousands of times, for each set of parameters (with and without modification): DBLP: 2,000, Wiki Talk: 5,000, US. Patent: 200,000. Such large numbers are needed in order to allow a high resolution histogram since many decisions of nodes in the model are probabilistic.

#### Simulation formulas

The parameter describing the flow of information in the network is the probability of consuming information, which depends on the tie strength between two nodes and is proportional to the intensity of the connection. Tie strength in the Wiki Talk network is inferred from the number of edits, in the Patent network from the number of citations and in DBLP from the number of co-authored papers. For each network the probability for consuming information is calculated in a manner that reflects either weak or strong ties and is based on the assumption that information received from a strong tie will be consumed with higher probability than that received from a weak tie. As each network has unique direction, degree and tie strength distribution, we performed sensitivity tests and determined the probabilities of consumption to be the lowest probability for a diffusion process in that specific network up to reaching super spread. A super spread is defined as a situation in which at least 20% of the nodes in the network consume the information.

Table 2 outlines the probabilities of information consumption per network when information is shared via weak and strong ties.

**Table 2:** Probabilities for information consumption via weak and strong ties.

Network	Weak ties	Strong ties
DBLP	0.15	0.38
Wiki Talk	0.20	0.50
US Patent	0.04	0.08

In addition to tie strength, consumption is related to the value of information. To allow comparison between consecutive simulations, the initial value of information is set to 1 (denoted by the letter V). The value of information changes during its spread due to modification activity. The following formula shows how value influences the probability of information consumption.

$${P_{Consumption}:\;1-(1-P_{TieStrength})}^{V}$$ (1)

In accordance with CMT, the value of information is affected by the modifications it undergoes. Modifications can be either constant (decelerating production function, i.e., AM) or relative (accelerating production function, i.e., RM).

$$AM:\;V_{new}=V\pm 0.2$$ (2)

$$RM:\;V_{new}=V\pm 0.2V$$ (3)

A 20% change in the value of information is chosen to reflect a change that is "modest" and realistic, that is, neither negligible (e.g. 5%) nor too dominant (e.g. 75%). The simulation randomly assigned a positive and a negative change with a 50% chance, to prevent bias. This assumption could easily be relaxed, to allow the study of asymmetric diffusion processes, but we leave this to future research.

#### Probability of sharing information

According to Allsop, Bassett & Hoskins [25] there is a 60% willingness to share information in social networks. We combined this basic probability in our formula. We assume that modifying information enhances the inclination to share it compared to sharing unmodified information because from a subjective point of view, modified information has a higher value as a node may view the modification as a benefit or as a result of personalization. The formula we implemented is based on the same logic from probability theory as P_consumption_ above. It regards the probability to share information as higher when reinvention occurs, even when it lowered the value of information (new value, i.e., V_new_). In the simulation, we take the absolute value of the RI modification.

$$V_{new}=V+0.2V\;or\;V_{new}=V+0.2\;for\;RRI\;and\;ARI\;respectively$$ (4)

Willingness to share = 60%

$$P_{Sharing}=1-{\left(1-willingness\;to\;share\right)}^{Vnew}$$ (5)

#### Probability of modification

The chance to modify information is calculated independently for each consumer. According to Rogers, Innovators comprise 2.5% of the social system. One option we considered was to allow only the first 2.5% of consumers (the innovators) to do all the modification and then stop it completely. However, based on Rogers' categories, we assigned the innovators a 25% chance to modify and a 2.5% probability of modification to the rest of the nodes.

$$P_{mo}=25\%\;in\;the\;first\;5\;steps;2.5\%\;in\;the\;following\;steps$$ (6)

Modifications added to or subtracted from V on a 50% chance basis.

Table 3 summarizes the model formulas for information's value and the consumption, sharing and modifying probabilities.

**Table 3:** Model Formulas.

Parameters	Formula
Probability of consumption	*P*_*Consumption*_: 1 − (1 − *P*_*TieStrength*_)^*V*^
Information value: relative change	*RM*: *V*_*new*_ = *V* ± 0.2*V*
Information value: constant change	*AM*: *V*_*new*_ = *V* ± 0.2
Probability of sharing information	*P*_*Sharing*_ = 1 − (1 − *willingness to share*)^*Vnew*^
Probability of modification	*P*_*mo*_ = 25% *in the first* 5 *steps*; 2.5% *in the following steps*

### Variable Description

#### Independent variables

**Modification:** Positive or negative modification of original information

**Relative modification (RM):** Adding or subtracting 20% from the value of information with each modification, simulates an accelerating production function.

**Absolute modification (AM):** Adding or subtracting a constant value set to 20% of the initial value of information at the starting point. This simulates a decelerating production function

**Tie strength:** Inferred from the frequency of joint activity [33, 34, 49]

**Degree:** Represented by average node links [50]

**Network directionality:** Directed or undirected

#### Dependent variables

**Critical mass:** Average number of nodes sharing information until an inflection point is reached

**Information reach:** Average number of nodes receiving information at the end of the diffusion process

**Sustainable diffusion:** Number of iterations following the inflection point until termination

**Collective outcome:** Total value of information normalized per number of nodes that have consumed it

## Results

Table 4 shows the percentage of nodes in each network that received, consumed, modified and shared information.

**Table 4:** Percentage of nodes receiving, consuming, modifying and sharing information.

Network	Received & did not consume	Consumed & did not share	Shared information	Modified & did not share	Modified & shared
**DBLP**	78	6.5	10	2	3.5
**Wiki Talk**	79	7.5	10	1.5	2
**US Patent**	52	14.3	21.7	4	8

The finding that in 52–78% of the cases information is not consumed by receiving nodes strengthens our notion that exposure to information is not enough for it to spread. The low percentage of cases in which modified information is shared is surprising considering the effect modification has on overall diffusion, as will be explained further on.

Rogers [38] defined the inflection point as the point in time in which the rate of adoption is fastest, i.e. the number of new adopters is increasing most rapidly. He further identified the inflection point occurs when approximately 16% of the social system has adopted an innovation. Following Rogers' definition, we measured the percentage of nodes required to reach the point in which the number of new adopters is increasing most rapidly. This is realized by comparing situations in which no modification takes place (NM), when modification is assigned a relative change in value (RM) and when modification is assigned a constant change in value (AM).

Table 5 compares between the percentage of nodes involved in reaching inflection point in each of the networks analyzed in accordance to the type of modification and the tie strength between nodes sharing information.

**Table 5:** Percentage of network nodes involved in reaching inflection point.

	No Modification		Relative Modification		Absolute Modification	
	Weak ties	Strong ties	Weak ties	Strong ties	Weak ties	Strong ties
**DBLP**	5.8	19.67	9.6	21.54	8.7	21.76
**Wiki Talk**	3.99	14.78	4.72	15.28	4.60	15.41
**US Patent**	0.3	3.4	0. 34	3.88	0.34	3.88

Interestingly, an inflection point occurs *sooner* in the process than the 16% anticipated by Rogers. In the No Modification (NM) scenario, an inflection point occurs on average when 3.3% of nodes are involved in the process when shared via weak ties, and when shared via strong ties, 12.6% of nodes. In the Relative Modification (RM) scenario–when transmitted via weak ties, on average 4.9% of nodes are involved in the realization of the inflection point, and 13.6% when transmitted via strong ties. Looking at the Absolute Modification (AM) scenario, 4.5% of nodes on average are involved when information is shared via weak ties, and 13.7% when shared via strong ties. Proposition 1(a) is supported together with an unexpected result–in all three networks, modification *delayed* reaching an inflection point as a higher percentage of nodes are involved in the diffusion process when information is modified.

Regarding Proposition 1(b), the effect of modification on reaching the inflection point is stronger in the undirected network, but in a negative sense. The percentage of nodes required to reach the inflection point in the undirected network, DBLP, is 1.6 times higher when modification takes place, while only 1.1 times higher in the directed networks, Wiki Talk and US Patent. Proposition 1(c) is accepted as well—as the realization of the inflection point is affected by the tie strength.

**Fig 3:**
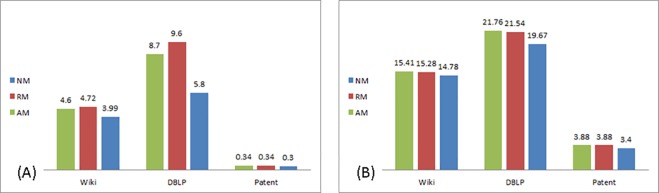
Percentage of nodes to inflection point. (A) Weak ties. (B) Strong ties.

The percentage of nodes involved in the realization of the inflection point is presented in Fig 3A and 3B for the weak and strong tie scenarios, respectively:

Looking at the interplay between tie strength, directionality and modification we found that in DBLP (undirected) and Wiki Talk (directed) the type of modification interacts with tie strength in a similar manner–in weak ties, relative modification (RM) had a stronger effect. In strong ties–the absolute modification (AM) had a stronger effect. This difference implies that when transmitted via weak ties, modification done in subsequent stages of the diffusion process has a stronger effect on diffusion than that done in its initial stages. When transmitted via strong ties, it is the opposite–initial modification carries a more pronounced effect than modification in later stages of the diffusion process. No difference between the types of modification was found in the Patent network. This may be due to the overall smaller scope of diffusion in this network—less than 8% in the case of strong ties, and less than 1% when weak ties are transmitting information.

**Fig 4:**
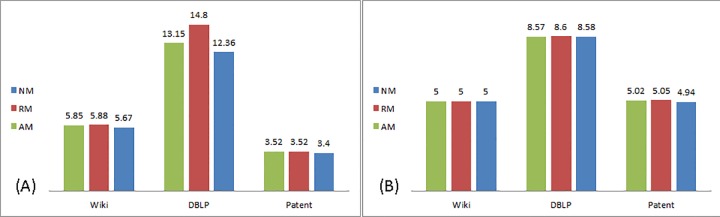
Number of iterations to inflection point. (A) Weak ties. (B) Strong ties.

The effect of modifications on the realization of the inflection point is analyzed by an additional measure: the number of iterations required for attaining this point in the diffusion process. Fig 4A displays the number of iterations to inflection point when information is shared via weak ties, and Fig 4B, when information is shared via strong ties:

As seen in Fig 4A and 4B, when shared via strong ties modification has no effect on the number of iterations in all three networks. Differences between the networks appear when information is shared via weak ties–in this case, the effect of modification on the number of iterations is negligible in the two directed networks and stronger in the undirected network, DBLP.

These findings suggest that the effect of information modification is influenced by the rate of diffusion. As diffusion is abrupt in strong ties, modifications occur mainly in the initial stages, and thus are more effective than later on. When transmitted via weak ties, diffusion is slower, allowing subsequent modifications to take place. In the Patent network the overall effect of modification is negligible as diffusion is smaller in scope.

Propositions 3 and 4 concern the effect of modifications on overall diffusion in terms of reach and sustainability. Reach is measured by the percentage of network nodes information has reached at the end of the diffusion process. Sustainability is inferred from the number of iterations required for information to reach a final spread in the network. The higher the number of iterations, the longer information propagates within the network, thus implying a sustainable process.

**Reach.** In all three networks, regardless of tie strength, modified information reached a higher percentage of nodes in the network than unmodified information. Comparing the networks reveals that the most pronounced effect of modifications is in the DBLP network which is undirected. In this network, when shared via weak ties, modified information reached 2.7 times more nodes than unmodified information. In the two directed networks modified information reached 1.2 times more nodes. Proposition 2a is accepted–the influence of individual behavior on the reach of information is affected by the directionality of the networks.

Regarding the effect of tie strength on diffusion, information shared via strong ties reached a higher percentage of nodes in the networks than that shared via weak ties. Interestingly, we found that when modifications of information take place, the difference in reach in strong and weak tie scenarios shrank, especially when it was modification of the relative type. In Table 6, the difference (in percentage) between reach in strong and weak ties is presented for the three situations: no modification takes pace (NM), relative modification (RM) and when modification is assigned an absolute value (AM). For example, in the DBLP network, in the NM condition, information shared via strong ties reached 3.1% more nodes than information shared via weak ties. When modifications took place, information shared via strong ties reached only 1.3% more nodes than when shared via weak ties.

**Table 6:** The difference (in %) between information reach in strong and weak ties.

	DBLP	WikiTalk	US Patent
**No M**	3.1	2.4	11.8
**Relative M**	1.3	2.1	11.6
**Absolute M**	1.9	2.1	11.6

Table 6 shows the difference (in %) of information reach between strong and weak ties in each of the networks analyzed in the three situations: when no modification occurs, when modification is assigned a relative and absolute value.

**Fig 5:**
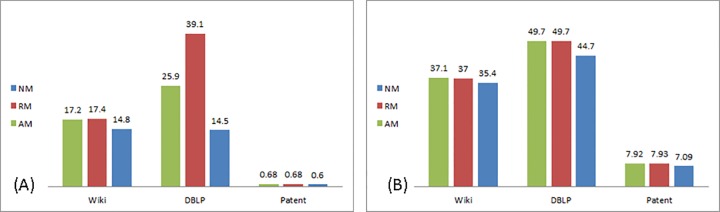
Percentage of nodes to final spread. (A) Weak ties. (B) Strong ties.

Fig 5A and 5B show that the effect of modifications on diffusion is stronger in weak ties compared to strong ties. Proposition 2b is accepted. Interestingly, tie strength interacts differently with the two modification schemes: in the DBLP network, when shared via weak ties, modification of the relative type (RM) have a larger effect, implying that modifications performed in late phases of the diffusion process have a bigger effect on reach than those performed in the initial stages. In the directed networks no substantial difference between the two modifications schemes is found. This finding will be elaborated in the discussion.

In order to further verify the effect of modifications on reach, we performed an additional analysis to determine the depth of reach. We were interested whether modified information transmitted via weak ties not only reached a higher percentage of nodes, but also nodes located in the periphery of the network.

Location is associated with degree: peripheral nodes have fewer connections, i.e. a lower degree measure than central nodes. The analysis was carried out by calculating the average degree of nodes involved in the diffusion process.

A comparison of the average degree of nodes at the end of diffusion is presented in Table 7. Across all three networks, the average degree of nodes is found to be smaller when modification took place, with smallest degree measurements for situations in which modifications of the relative type occurred (except for the Patent network in which no difference between the two value schemes is found). This finding implies that modification induces information to reach remote nodes in the network.

**Table 7:** Average node degree of nodes involved in the diffusion process.

Network	No M	Relative M	Absolute M
**DBLP**	3.3	2.7	2.5
**Wiki Talk**	2.42	2.20	2.19
**US Patent**	1.97	1.87	1.87

Table 7 presents the average node degree of nodes involved in the diffusion process in each of the networks analyzed in three situations: when no modification occurs and when modification carries a relative and absolute change in the value of information.

Note that the stronger effect of modification on reaching remote nodes is found in the undirected DBLP network. Summarizing Proposition 2: the structural properties of direction and tie strength influences individual behavior and result in varying degrees of information reach within the networks.

**Sustainability.** Sustainability is inferred from the number of iterations required for information to reach a final spread in the network. The higher the number of iterations, the longer information propagates throughout the network, thus implying a sustainable process.

**Fig 6:**
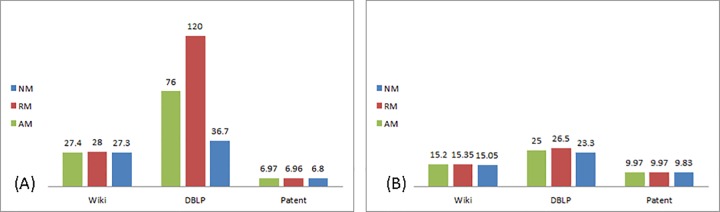
Number of iterations to final spread. (A) Weak ties. (B) Strong ties.

Fig 6A and 6B display the number of iterations to reach final spread when information is shared via weak and strong ties, respectively.

Regarding Proposition 3a, the effect of directionality on the sustainability of the diffusion process is evident: in the undirected network of DBLP the diffusion process is more sustainable than in the directed networks of WikiTalk and US Patent.

Modifications as a whole contributed to the sustainability of the diffusion process across all networks analyzed: modified information of both value change schemes propagated in the networks longer than unmodified information.

Considering Proposition 3b, the effect of modification is minor in the directed networks in both tie strengths, but is significant in the undirected network especially when assigned a relative value modification and transmitted via weak ties. In this case information propagated in the network up to 4 times longer than information that has not been modified.

When comparing the effect of tie strength an interesting phenomenon arises: in the DBLP and Wiki networks the diffusion process is more sustainable when information is shared via weak ties than in strong ties. In the Patent network, it is opposite.

In summary of Propositions 3 and 4, the contribution of modifications to the reach and sustainability of the diffusion process is stronger in the undirected network than in the directed ones. The interplay between tie strength and information modification is found mostly in the undirected network, in which its effect when shared via weak ties is prominent. In the directed networks the effect of modification on sustainability in both tie strengths is negligible. Modification with a relative change (RM) is evident in the undirected network, DBLP, especially when information is shared via weak ties. In both directed networks relative and absolute modification have a similar effect on reach and sustainability.

While Rogers [38] identified that reaching an inflection point as a determinant for self-sustained diffusion, we found self-sustainability to be determined much sooner than the inflection point. We term this point "the point of no return" which will be further elaborated in the Contribution to Theory section.

**Fig 7:**
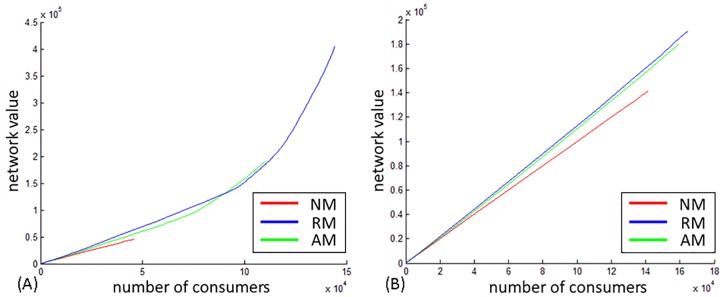
DBLP collective outcome. (A) Weak ties. (B) Strong ties.

**Fig 8:**
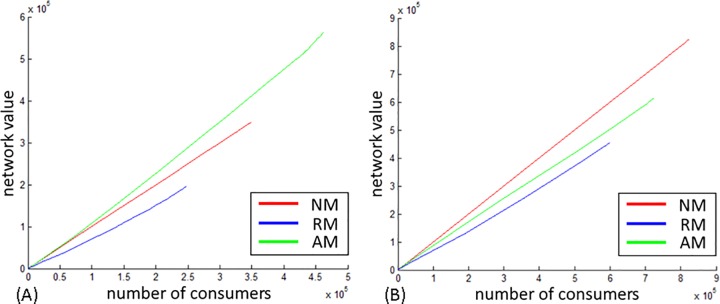
WikiTalk collective outcome. (A) Weak ties. (B) Strong ties.

**Fig 9:**
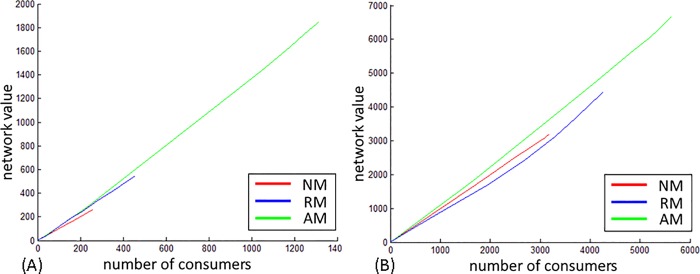
US Patent collective outcome. (A) Weak ties. (B) Strong ties.

**Collective outcome.** The spread of valuable information in a network is a desirable collective outcome for members of the network. As such, the contribution of modification to the value of information that diffuses in the network is of interest and is addressed in regard to directionality (P4) and tie strength (P5) by plotting the cumulative value of information that spreads in the network (Y axis) versus the number of consumers (X axis) in Figs 7–9.

In the DBLP, which is undirected, modifications enhanced the value of information in both tie strengths as indicated by the green and blue lines in Fig 7. The curve manifested in the relative value (RM) when information diffuses via weak ties indicates that modification happened repeatedly throughout the diffusion process and that late modifications have a greater contribution to the value of information than early modifications. This acceleration suggests that in undirected networks modified information transmitted via weak ties contributes to the overall diffusion of valuable information in the network.

In the WikiTalk network (Fig 8), modifications enhanced the value of information only when transmitted via weak ties. AM, indicated by the blue line implies that modifications performed in the initial stages of the diffusion process is of the highest contribution to the value of information.

In the Patent network, modifications contributed to the value of information in both tie strengths, with a stronger effect by initial modifications than by late modifications.

Referring to Proposition 4, in the context of the collective value of information, the most prominent difference between directed and undirected networks lies in the dynamics of modification activity. In the undirected network modifications happened repeatedly with subsequent nodes contributing more to the value of information than initial nodes, thus implying an evolutionary process for information throughout the diffusion. In the directed networks, modifications appearing in the initial stages of diffusion contribute more significantly to the value of information.

Regarding the interplay between modification and tie strength, Proposition 5 is partially supported, as the contribution of modifications to the value of information is greater when information diffuses via weak ties than via strong ties in both DBLP and WikiTalk networks, but does not have an effect in the US Patent network.

## Discussion

This study evaluated information diffusion dynamics by combining global network structural aspects with local decisions and actions taken by nodes.

Many widely used models describing diffusion of information are built on assumptions that, to our understanding, underestimate the complexity of the decisions taking place. Furthermore, models need to address the manner in which micro level behaviors aggregate to macro diffusion processes. By combining expressions from the DOI: communication channels (directionality), communication proximity (tie strength), individual behavior (modification of information) and the inflection point with CMT concepts: accelerating and decelerating production functions manifested in group processes, we address local (micro) and network (macro) points of view.

The challenge in designing the model was to address the diffusion of information in a manner that accounts for (a) modifications information undergoes throughout its spread; (b) the complexity of differentiating between consuming and sharing information; and (c) structural properties. These challenges are addressed by the proposed model: Consume, Modify, Share (CMS).

The model was applied to the graph of three actual networks harvested from the web, thus providing a means to test information flows in a realistic topology. The networks analyzed were the DBLP network of research scientists, Wiki Talk network, and the US Patent citation network. As these networks vary in structural properties such as direction, size, mean degree and node-link ratios, they provide diverse sources of data that may enrich our understanding of the interplay between structural properties and individual decisions and their effect on diffusion of information in networked settings.

The finding that in 52–79% of the cases information is not consumed by the receiving nodes (Table 4) supports our notion that diffusion of information is a complex phenomenon that cannot be attributed to mere exposure (contagion). Interestingly, the overall effect of the individual decision to modify information on the sustainability and reach of information in the networks is striking when considering the low percentages (2%-8%) of nodes that consume, share and modified information. This effect lends support to the choice of information modification as a relevant variable in diffusion studies. Although modification occurred rarely, it has a positive effect on the value of information propagating in all networks studied, i.e., on the collective outcome, implying that micro behavior, even when it has a negative value, may accumulate to a positive effect on the macro level. This finding is in accordance with Schelling's [51] observation that micro-motives aggregate to unpredictable macro-behaviors.

### The Interplay between Modifications of Information and Directionality

In directed networks spread and reach of information is limited compared to undirected structure [30]. Our results support previous findings regarding the effect of directionality on diffusion of information. In DBLP, which is undirected, unmodified information propagated in the network for a considerably longer time and reached a higher percentage of nodes than in the directed networks of Wiki Talk and Patent. Interestingly, differences were found between the two directed networks: in the Patent network information reached 7% of the network while in the Wiki Talk network information sharing reached 35% of the nodes (50% in the undirected DBLP). Both directed networks are less supportive of information sharing than the undirected network, with the Patent network the least supportive of information sharing.

As diffusion progresses the effect of directionality on diffusion processes increases. In the initial stages of diffusion, up to the inflection point, directionality has a minor effect. In later stages of diffusion, when social dynamics evolve, the effect of directionality increases.

Regarding the interplay between modifications and directionality: up to the inflection point, regardless of directionality, modification has a minor effect on the number of iterations needed and percentage of nodes involved in the process. It is in the longer process of reaching full spread that differences between network types become evident. In this process, two parameters were evaluated: sustainability (the number of iterations required to attain full spread) and reach (the percentage of networks information has reached). Regarding sustainability, the effect of modification on the number of iterations to final spread was minor in both network types when information was shared via strong ties. When shared via weak ties, the difference between directed and undirected networks was robust. In the undirected network, there was a 227% growth in the number of iterations when modifications occurred in weak ties, as opposed to 2.4% in the directed networks.

Regarding reach, in the directed networks, modification is found to have a negligible effect on reach, with no distinct difference between the two value schemes assigned to modifications. In the undirected networks, the effect of modification on reach is pronounced, especially when information is shared via weak ties and assigned a relative value.

A note regarding the US Patent network is in order. Based on the temporal nature of citing, in which patents can only cite earlier patents, all edges in the network point backwards, thus forming a directed, temporal, acyclic graph. In this network, diffusion is limited: unmodified information reaches 7% of the network when shared via strong ties and less than 1% when shared via weak ties. Although modifications took place in 8% of the cases–the highest percentage of all three networks studied–it had a negligible effect on diffusion, with no difference between relative and absolute value schemes assigned to modified information. The combination of the direction and temporality thus appears to be unfavorable for information diffusion.

When connections in the network are asymmetrical (directed networks), social benefits such as building social ties, prestige and influence, may be achieved by publishing original information, and not so much by the cooperation and dialogue that modification affords. Symmetrical connections (undirected networks) built on reciprocity and dialogue serve for building social ties, prestige and influence through conversation. Modifications appear to be a type of "conversation" that is appreciated in this network type: modified information propagated in undirected networks for a considerably longer time and reaches isolated nodes that otherwise would not have been involved. Thus, the underlying connectivity patterns that directionality entails drive the social aspects of communication.

### The Interplay between Modifications of Information and Tie Strength

Analyzing the interaction between tie strength and the act of modifying information yields interesting observations. In the literature, tie strength is associated with the *type* of information, i.e., weak ties play a role in transmitting unique and novel information [35, 52]; the *speed* of diffusion, which is associated with strong ties; and the *reach* of diffusion, which is associated with weak ties [30, 42, 43, 53]. Regarding the type of information, modification is of itself a unique type of information and the CMS model enabled the study not only of the type but also the value of information that diffuses in the networks. The value of information is inferred from the collective outcome. Interestingly, when modifications occur, strong and weak ties disseminate information of higher value. However, it is through weak ties, especially in the undirected network, that the value of modified information is the highest. These ties are "responsible" for repeated modifications. Not only do weak ties transfer novel information, but the dynamics of diffusion within them promotes creativity and contributes to the value of information as well.

### Sustainability and Reach

While DOI concentrates on the rate of diffusion, CMT focuses on the preconditions for sustainability. This work adopted the latter emphasis and accordingly tended to focus on sustainability, which is inferred from the number of iterations required for information to reach full spread. Findings show that weak, rather than strong ties contribute to the sustainability of the diffusion process as information transmitted by the former remains longer in the network. When modification occurs, the contribution of weak ties grows substantially, but only in undirected networks.

Turning to reach, present findings contradict previous findings in which weak ties are associated with reach. Across the networks studied, information transmitted via strong ties reached a higher percentage of nodes than that shared via weak ties. As seen in Table 6, when modification occurred, the difference between the percentages of nodes reached in strong and weak tie scenarios shrank, especially for relative modification.

Looking at the breadth of diffusion, modified information shared via weak ties is found to have a pronounced effect. This finding emerges by calculating the average degree of nodes involved in the diffusion process with and without modifications. Degree is associated with location: peripheral nodes are less connected and, naturally, have a lower degree count than central nodes in the network. As seen in Table 7, regardless of direction, when modifications take place the mean degree of nodes is smaller than when information is shared without modifications. Furthermore, the smallest degree measurements are found in situations in which relative modification occurs. Thus, modification is a mechanism that reaches and engages parts of the network that would not have been involved or informed otherwise.

In addition, an interesting interplay between tie strength and the type of modification taking place surfaced: when transmitted via strong ties, modification of the absolute type carries a stronger effect, and when transmitted via weak ties, it is modification of the relative type that is stronger. This holds true for reach measurements throughout the diffusion process: up to, and following the inflection point. This interaction suggests that in the strong tie scenario the initial nodes performing modification that have a stronger effect on reach, and in the weak tie scenario it is modification performed by subsequent nodes that has more impact. It is through weak ties, then, that diffusion accelerates to the degree required for sustainable diffusion.

The absolute and relative value change schemes assigned to modified information simulate negative and positive interdependence dynamics between nodes. Absolute value (AM) simulates negative interdependence, decreasing the rate and likelihood of contributions. Relative value (RM) simulates positive interdependence. The negative interdependence found when modified information is shared via strong ties implies that the initial modification has the greatest effect on the value of information, while subsequent nodes have an incremental effect. This supports the observation that strong ties are homophilious, while weak ties are heterophilious in nature [34, 54]. Thus, when communicating with homophilious "like-minded" others, the added value of subsequent modifications has a diminishing effect. The positive interdependence found when weak ties are involved in modifying information supports the notion that communication within a heterophilious group of diverse backgrounds enriches the conversation and makes each contribution more worthwhile.

### Conditions Necessary to Maximize the Effect of Modifications

Modifying information has a positive contribution to the diffusion process in terms of sustainability and reach. The most pronounced effect of modifications is found in the undirected networks when information is shared via weak ties and assigned a relative value. This finding contributes to identifying the primary conditions required for modifications to have a substantial effect to be *duration* and *reciprocity*.

Regarding duration, for modifications to be effective a long diffusion process is needed. Longer diffusion allows higher incidence of modifications promoting the evolutionary process that makes information more attractive to a greater variety of nodes in the network. Diffusion is longer in undirected networks enhancing the effect of modifications. In addition, weak ties slow down diffusion explaining the stronger effect modification has when shared via weak ties. The stronger effect of relative modification suggests that the slower rate of diffusion in weak ties allows information that is modified to undergo an evolutionary process in which it enhances the value of information that spreads in the network, with subsequent nodes being more influential on attaining higher value than initial nodes.

Regarding the second condition, reciprocity is a key concept for understanding social interactions [55]. Reciprocal exchange is motivated by amassing prestige [56, 57], sharing interest and promoting human relationships [58]. In the context of networks, reciprocity is associated with communication type and is tied to the concept of interactivity that describes the extent to which messages in a sequence relate to each other [59]. Reciprocity occurs more frequently when edges are symmetrical (e.g., Facebook friends) and less when they are asymmetrical (e.g., Twitter followers). Kwak, Lee, Park and Moon [60] found that only 22.1% of the users in Twitter, a directed network, have reciprocal relationships, and that interactions are mostly motivated by search for information as opposed to facilitating relationship formation as in Facebook [61]. The substantial effect of modifications in undirected networks as opposed to its limited effect in directed networks can be attributed to the reciprocal communication in undirected networks that is relatively lacking in directed networks.

#### The contribution of modifications to the collective outcome

In all three networks, when shared via weak ties, modifications enhanced the value of information that spread throughout the network. While modifications occurred in the directed networks at the beginning of the diffusion process and then ceased (indicated by the straight line in Figs 7–9), in the undirected network, modifications repeated throughout diffusion, as manifested by the curve in the relative modification scenario. This curve indicates that repetitions enhanced the value of information. The value of information grew with the number of consumers and subsequent modifications had the greatest effect on the collective outcome. This acceleration suggests that in undirected networks modified information transmitted via weak ties contributes to the overall diffusion of valuable information in the network. In directed networks modifications performed in the initial stages of diffusion contribute to the value of information, while in undirected, contribution comes from modifications performed in subsequent stages.

In summary, we found the effect of modification to vary throughout the diffusion process: while having a negative effect on reaching the inflection point, by delaying its realization, modifications carry positive effect on overall diffusion across all variables: the lower average node degree when modifications take place, the higher percentage of the network reached by modified information, the longer propagation in the network, the higher value of modified information in the network–all imply that modifications contribute to the diffusion of information in the network in reach and sustainability. Although the effect of modification is manifested in directed and undirected networks, it is stronger in the undirected network.

### Contribution to Theory

Beyond corroborating known phenomena (strength of weak ties) the CMS model offers new knowledge on information diffusion and the special role of the decision to modify information. In addition, we identified nuanced observations regarding Rogers' assertions about the inflection point and the manners in which the CMS model contributes to Critical Mass Theory. In the following we unpack these two contributions.

#### Contribution to the Diffusion of Innovations Theory

The definition of the inflection point and its relevance to a sustainable diffusion: in the case of digital networks, our finding questions the identification and contribution of the inflection point to the diffusion process as introduced by Rogers [38]. Tie strength and directionality of the networks influence the point in the diffusion process in which the rate of the adoption is fastest (Table 5). These findings highlight the effect of direction on reaching inflection point and the need for measures that consider direction. Moreover, these findings suggest that different types of innovation diffuse through networks in different patterns, i.e., the diffusion of information differs from the diffusion of tangible innovations.

The inflection point was identified by Rogers as a determinant for diffusion to become self-sustained. We found the diffusion process became self-sustained as soon as a hub shared information and termed this as "the point of no return". Identifying the "point of no return" spun our attention to the definition offered by CMT regarding "critical mass"—the identification of the initial group of nodes needed for the diffusion process to become self- sustained.

CMT defines the critical mass as the "small segment of the population that chooses to contribute to the collective action thus creating conditions for the majority to join leading to the achievement of the collective good" [7]. When looking at this initial, small segment, we find it to be different from the segment identified by the DOI. In the DOI, this segment comprises the nodes involved in the diffusion process up to inflection point–and is built upon innovators (2.5%) and early adopters (13.5%). Our findings show that in the case of information diffusion innovators ***alone*** can comprise the critical mass needed for attaining vast diffusion, as long as they are friends of hubs.

This finding is in accordance with Dezso & Barabasi's [62] observation that in scale free networks epidemic thresholds vanish as a consequence of the existence of hubs. In other words, the presence of neighboring hubs, rather than the number of nodes, is crucial for diffusion. The conditions necessary for sustainable diffusion in networks are structurally and not quantitatively dependent.

#### Contribution to Critical Mass Theory

Critical Mass Theory emphasizes the role played by heterogeneity of resources and interest levels on attaining the critical mass of initial participants in a diffusion process. CMT has been criticized on three main grounds: (a) The simulations it is built upon mainly address situations involving an accelerating production function, thus failing to capture the complexity of situations that entail decelerating production functions [63]; (b) These simulations are designed with one-step mobilization, meaning that organizers mobilize only direct ties and those in which individuals possess perfect information regarding other's doings [42, 64]; and (c) CMT treats contributions as substitutional, i.e., the relative weights of contributors are not taken into consideration [65]. According to Witzel, Beimborn and König [66], the lack of concern for individual variance weakens the applicability of standardization models. This criticism is addressed in the present research by implementing the variance in participant contributions in accounting for the varying tie strength and degree measures and incorporating multiple steps of mobilization.

The present research addresses these points. We demonstrate the theory’s applicability in a multi-step diffusion process with accelerating and decelerating production functions, while taking tie strengths, i.e., relative weights of contributors, into due consideration.

Applying the dynamics of production functions to the diffusion of information in networks contributes to the understanding of the dynamics associated with sharing information. As production functions imply interdependence that can be positive (accelerating) or negative (decelerating), they can be used to infer node behaviors in networks and manifest the manner in which node-related behaviors aggregate into global network effects. For example, the interaction found between type of modification (absolute, i.e., decelerating and relative, i.e., accelerating) and tie strength sheds light on communication patterns in social networks.

Adapting CMT to the diffusion of information in networks broadens the theory's usage and theoretical implications. Adapting its main constructs to those used in social network analysis–for example, using degree as a surrogate measure for resource and using tie strength as a surrogate measure for interest–opens new opportunities for contemporary studies that rely on CMT.

#### Contribution to Modeling Diffusion

Diffusion of information is a complex phenomenon that involves, at the actors' level, a two- stage decision process: the decision to consume information and the decision to share it with others. It is through retransmissions (re-sharing) performed by network nodes that information diffuses. Both stages are influenced by an array of factors that involve image [27], communicator’s identity [20] and adding value to one's social circles [67], to name a few. Content properties are yet another factor influencing the diffusion of information, as outlined in the theoretical background section. At the network level the diffusion of information is influenced by global structural properties such as directionality and the tie strength between nodes.

Going back to diffusion as synonymous with contagion, as suggested by epidemiological models such as the Susceptible-Infected-Susceptible Model [4] and the Independent Cascade Model [9, 10, 68], the decision process involved in consuming and sharing information and the content aspects informing these decisions are largely overlooked. On the other hand, models that do relate to the decision-making process involved, such as the Linear Threshold Model [12], assume that the decision to adopt depends on whether the number of a member's adopting neighbors exceeds some personal threshold and focus on the diffusion of influence [9] associated with the adoption of tangible products.

In modeling diffusion, the unique properties of information need to be addressed. These include the ease in which it can be shared (especially in online environments), the subjective value it carries [69], and the manner in which it is prone to modifications [18, 19]. Common models of diffusion overlook these properties of information and treat it as invariant during the diffusion process.

Furthermore, information diffusion models view the decision to adopt or consume information to be binary: nodes are either "infected" or not, either "adopt" or not. Treating information as prone to modifications widens the possibilities for potential adopters, thus allowing a middle way–to consume information and share with modifications, as common in today’s prevalent participatory culture [41].

The CMS Model introduced here contributes to existing models in that it encompasses the actor, content and structure components that influence the diffusion of information. By combining all three, the dynamics involved in diffusion processes are addressed at the personal and global levels, offering preliminary insights into the way network structure affects the behavior of nodes.

The finding that modification involves positive interdependence between weak ties and negative interdependence when shared via strong ties sheds light on factors that contribute to the sustainability and reach of the diffusion process. In addition, the model reveals that modifications have a varying effect throughout the diffusion process: negative up to the inflection point, and positive effect on overall diffusion. This finding turns attention to the role subsequent stages of the diffusion process play in the success of diffusion, which opens new opportunities for the study of maximizing diffusion. Designed to address the unique properties of information, the CMS model may be used to study additional variables associated with the diffusion of information such as social influence, interactivity, WOM dynamics, gender inclinations, etc.

### Limitations and Future Work

The CMS model presented here was designed with best intentions to simulate real world decision making regarding the spread of information in networks. Still, the model suffers from some limitations. Starting with simplification, by synthesizing the variables into a single numeric value our results point out general directions or trends and not absolute values. In our analysis we took care to discuss relative outcomes, but not absolute values.

In addition, we followed Roger's observation of innovators comprising 2.5% of the network and assigned innovators randomly, not by their structural properties in the network. In future work, it will be interesting to align network structure and attributes associated with the specific innovativeness levels.

Our model allowed nodes to receive information once, implying that the effects reported here are probably conservative. Further research may incorporate the opportunity of multiple possibilities to receive and consume information. Additional and varied starting points as well as multiple simultaneous starting points should be examined to further understand the effect of network structure on the diffusion of information. Future research will address the interplay between modifications and additional structural properties such as density and clustering coefficient. Lastly, studying three networks gave us a rich mosaic of observations, but our findings are not "universal" therefore more networks need to be researched.

## Supporting Information

S1 Dataset DBLP_strong_sizes_of_critical_mass_groups_Table 5. (TXT)

S2 Dataset DBLP_weak_sizes_of_critical_mass_groups_Table 5. (TXT)

S3 Dataset Patent_strong_sizes_of_critical_mass_groups_Table 5. (TXT)

S4 Dataset Patent_weak_sizes_of_critical_mass_groups_Table 5. (TXT)

S5 Dataset WikiTalk_strong_sizes_of_critical_mass_groups_Table 5. (TXT)

S6 Dataset WikiTalk_weak_sizes_of_critical_mass_groups_Table 5. (TXT)

S7 Dataset DBLP_strong_degrees_of_consumer_nodes_groups_Table 7. (TXT)

S8 Dataset DBLP_weak_degrees_of_consumer_nodes_groups_Table 7. (TXT)

S9 Dataset Patent_strong_degrees_of_consumer_nodes_groups_Table 7. (TXT)

S10 Dataset Patent_weak_degrees_of_consumer_nodes_groups_Table 7. (TXT)

S11 Dataset WikiTalk_strong_degrees_of_consumer_nodes_groups_Table 7. (TXT)

S12 Dataset WikiTalk_weak_degrees_of_consumer_nodes_groups_Table 7. (TXT)

S13 Dataset DBLP_weak_ties_value_vs_consumers_Fig 7A. (TXT)

S14 Dataset DBLP_strong_ ties_value_vs_consumers_Fig 7B. (TXT)

S15 Dataset WikiTalk_ weak_ties_value_vs_consumers_Fig 8A. (TXT)

S16 Dataset WikiTalk_strong_ ties_value_vs_consumers_Fig 8B. (TXT)

S17 Dataset Patent_ weak_ties_value_vs_consumers_Fig 9A. (TXT)

S18 Dataset Patent_ strong_ ties_value_vs_consumers_Fig 9B. (TXT)

S1 Table Experiment iterations summary. (DOCX)
