# Supplementary material for: Consume, Modify, Share (CMS): The Interplay between Individual Decisions and Structural Network Properties in the Diffusion of Information
Source: PLoS One. 2016 Oct 31;11(10):e0164651. doi: 10.1371/journal.pone.0164651 (PMC5087943; doi:10.1371/journal.pone.0164651)
Supplement: S1 Table — (DOCX) [file pone.0164651.s019.docx]

Summary of the average results for thousands of experiment iterations.

|  | No Modification | | Relative Modification | | Absolute Modification | | Supporting information |
| --- | --- | --- | --- | --- | --- | --- | --- |
| # of iterations to inflection point | Weak ties | Strong ties | Weak  ties | Strong ties | Weak ties | Strong ties |  |
| DBLP | 12.36 | 8.58 | 14.8 | 8.6 | 13.1 | 8.57 | S4 a &b Fig. |
| Wiki Talk | 5.67 | 5 | 5.88 | 5 | 5.85 | 5 | S4 a &b Fig. |
| US Patent | 3.4 | 4.94 | 3.5 | 5.03 | 3.52 | 5.02 | S4 a &b Fig. |
| # of nodes in inflection point | Weak ties | Strong ties | Weak  ties | Strong ties | Weak ties | Strong ties |  |
| DBLP | 18,600 | 62,400 | 30,500 | 68,300 | 27,600 | 69,000 |  |
| Wiki Talk | 95,700 | 354,000 | 113,000 | 366,000 | 110,000 | 369,000 |  |
| US Patent | 80.5 | 916 | 92 | 1046 | 91.9 | 1045 |  |
| % of nodes to inflection point | Weak ties | Strong ties | Weak ties | Strong ties | Weak ties | Strong ties |  |
| DBLP | 5.8 | 19.67 | 9.6 | 21.54 | 8.7 | 21.76 | S3a &b Fig. S5 Table |
| Wiki Talk | 3.99 | 14.78 | 4.72 | 15.28 | 4.60 | 15.41 | S3a &b Fig. S5 Table |
| US Patent | 0.29 | 3.4 | 0.34 | 3.88 | 0.34 | 3.88 | S3a &b Fig. S5 Table |
| # of iteration to reach final spread after inflection point | Weak ties | Strong ties | Weak ties | Strong ties | Weak ties | Strong ties |  |
| DBLP | 36.7 | 23.3 | 120 | 26.5 | 76 | 25 | S6a&b Fig. |
| Wiki Talk | 27.3 | 15.05 | 28 | 15.35 | 27.4 | 15.2 | S6a&b Fig. |
| US Patent | 6.8 | 9.83 | 6.96 | 9.97 | 6.97 | 9.97 | S6a&b Fig. |
| Probability of super-spread (%) | Weak ties | Strong ties | Weak ties | Strong ties | Weak ties | Strong ties |  |
| DBLP | 17 | 40 | 16.3 | 41.4 | 16.6 | 40.3 |  |
| Wiki Talk | 11.7 | 29.7 | 12.7 | 30.5 | 12 | 31 |  |
| US Patent |  |  |  |  |  |  |  |
| Percentage of network reached | Weak ties | Strong ties | Weak ties | Strong ties | Weak ties | Strong ties |  |
| DBLP | 14.5 | 44.7 | 39.1 | 49.7 | 25.9 | 49.7 | S5ab Fig. S6 Table |
| Wiki Talk | 14.8 | 35.4 | 17.4 | 37 | 17.2 | 37.1 | S5ab Fig. S6 Table |
| US Patent | 0.6 | 7.09 | 0.68 | 7.93 | 0.68 | 7.92 | S5ab Fig. S6 Table |
